# Probabilistic Estimation of Dietary Intake of Methylmercury from Fish in Japan

**DOI:** 10.14252/foodsafetyfscj.D-20-00018

**Published:** 2021-02-10

**Authors:** Takahiro Watanabe, Rieko Matsuda, Chikako Uneyama

**Affiliations:** 1 Food Safety Information Division, National Institute of Health Sciences, 3-25-26, Tonomachi, Kawasaki-ku, Kawasaki, Kanagawa 210-9501, Japan

**Keywords:** methylmercury, dietary intake, probabilistic estimation, Monte Carlo simulation

## Abstract

Dietary intake of methylmercury from fish was estimated via Monte Carlo simulation using data for methylmercury concentrations in 210 fish samples and data regarding fish consumption extracted from the Japanese National Health and Nutrition Survey. The fish analyzed were classified into 5 groups according to categories used in the survey. The distribution of consumption of fish from each group was used without fitting to statistical distributions. A log-normal distribution was fitted to the distribution of methylmercury concentration in each fish group. Two random numbers that followed these distributions were generated, and a trial value was calculated by multiplying these random numbers. The trial value was divided by the body weight (50 kg) to arrive at an estimate of dietary methylmercury intake. A total of 100,000 Monte Carlo simulation iterations were performed. The estimated mean daily intake of methylmercury was 0.093 µg/kg body weight (bw)/day. This value is well below the tolerable daily intake of 0.292 µg/kg bw/day calculated from the tolerable weekly intake (2.0 µg/kg bw/week) established by the Food Safety Commission of Japan. The probability that the daily intake of methylmercury exceeds the tolerable daily intake was 7.6%. As there are no data regarding fish consumption for consecutive days, estimation of the weekly intake of methylmercury is a subject for future studies.

## 1. Introduction

Mercury occurs naturally and is distributed throughout the environment by both natural process and human activities. Since “Minamata disease” emerged in the 1950s, mercury has attracted worldwide attention and is considered by the World Health Organization (WHO) as a top 10 chemical or group of chemicals of major public health concern^1^^)^. Mercury exists in various inorganic and organic forms in nature. Methylmercury is a type of organic mercury that is neurotoxic and accumulates in higher trophic animals throughout the food chain^2^^)^. Humans are most susceptible to the adverse health effects of methylmercury during the fetal stage of development^3^^,^^4^^,^^5^^)^. After evaluating the risks of methylmercury, the WHO and other international organizations concluded that although the adult population does not face a significant health risk, the fetus is at risk. Based on the results of 2005 a food safety risk assessment in Japan, the Food Safety Commission of Japan established a methylmercury tolerable weekly intake (TWI) of 2.0 µg/kg body weight (bw)/week in women who are or may become pregnant^6^^)^.

Normally, fish consumption is considered the primary route of methylmercury intake in humans^7^^,^^8^^,^^9^^)^. Japan’s Food Sanitation Act imposed provisional restrictions of 0.4 mg/kg for mercury and 0.3 mg/kg for methylmercury (as mercury) on all fish and shellfish other than tuna, deep-sea fish and shellfish, and inland river fish and shellfish^10^^)^. To further mitigate risks associated with methylmercury, the Ministry of Health, Labour and Welfare (MHLW) provided guidance for fish consumption by women who are or may become pregnant, indicating recommended consumption amounts for different types of fish^11^^)^. The MHLW also prepared an information leaflet for the women in order to convey appropriate advice on fish consumption in an easy-to-understand manner^12^^)^.

In our total-diet study employing a market basket (MB) approach, the daily intake of methylmercury by the general Japanese population with average dietary habits was estimated at 0.132 µg/kg bw/day in 2015^13^^)^. The tolerable daily intake (TDI), calculated based on the TWI for convenience, is 0.286 µg/kg bw/day. According to these figures, methylmercury intake as a proportion of TDI is 46%. This intake determined using the MB approach is the mean for the general population of Japan. Given that this mean is approximately 50% of the TDI, those who ingest large amounts of methylmercury (toward the upper bound of the distribution) may be taking in an amount near or exceeding the TDI. In addition to mean intake, the upper limit or different percentiles of the distribution range must be determined to better evaluate the risk from methylmercury exposure. The MB approach is unsuited to determining intake distributions. Estimating intake via a duplicate-diet study or using a Monte Carlo simulation or other probabilistic approach is necessary. Estimating intake via a Monte Carlo simulation requires data regarding the concentrations of the target substance in individual food products and data indicating the distribution of the consumption of these food products. The intake contribution of the fish group to the total dietary intake of methylmercury estimated using the MB approach in 2015 was significant (90%), and these results suggest that fish are the primary source of methylmercury exposure for the general Japanese population. It should therefore be possible to estimate valid methylmercury intake values via a probabilistic approach using the distribution of methylmercury concentrations in fish and fish consumption data.

The authors previously developed a versatile gas chromatography–mass spectrometry method for determining methylmercury levels following phenylation^14^^)^. The method was improved by adding a pretreatment step to prevent losses in recovery from certain fish species, and the performance of the method was then evaluated^15^^)^. From 2014 to 2015, we used this method to determine total mercury and methylmercury concentrations in 210 samples from 19 species of fish sold as food^16^^)^. Our investigation found high methylmercury concentrations in large predatory fish such as swordfish, tuna, and wild yellowtail and low concentrations in smaller fish such as horse mackerel, pacific saury, and sardines. We also found total mercury concentrations to be closely correlated with methylmercury concentrations. The ratio of methylmercury expressed as mercury to total mercury ranged from 0.6 to 0.9, regardless of the total mercury concentration, in fish samples containing at least 0.1 mg/kg of total mercury.

Thus, the abovementioned data can be used to estimate the distribution of methylmercury concentrations in species of fish widely consumed in the Japanese diet. This report describes our attempt to probabilistically estimate the intake of methylmercury from fish by conducting a Monte Carlo simulation using methylmercury concentrations in widely consumed fish species and data regarding the distribution of fish consumption extracted from the Japanese National Health and Nutrition Survey^17^^)^.

## 2. Materials and Methods

### 2.1 Monte Carlo Simulation

Data on individual food consumption for men and women between the ages of 1 and 106 included in the results of the Japanese National Health and Nutrition Survey conducted from 2008 to 2010 was obtained through official application based on Statistics Act^18^^)^. The obtained data was protected by the Act and it was properly discarded after use. In the Survey, the consumption data of fish and shellfish is classified into the two categories of “raw fish and shellfish” and “processed fish and fish products”, which are further divided into 13 groups. The consumption data on 5 raw fish groups included in the 13 groups were used to estimate methylmercury intake. The 5 fish groups were horse mackerel/sardine group, salmon/trout group, red snapper/flounder group, tuna/swordfish group, and other raw fish group. Datasets extracted from the Survey (which contains 28,706 data points distinguished by fish species) were used as values for raw fish consumption by the general Japanese population. The data for each fish group had a high proportion of people who didn’t consume the fish group on the day of the survey, which rendered fitting to a standard continuous distribution impractical. We therefore arranged the data into a frequency distribution without fitting to statistical distributions. It may be useful to note that the mean value of the consumption of processed fish and fish products was 0.25 times lower than that of raw fish.

Methylmercury concentrations were determined in 210 samples of the following 19 species of fish: horse mackerel, sardine, mackerel, salmon, saury, rainbow trout, flounder, red snapper, cod, swordfish, bluefin tuna, big eye tuna, yellow fin tuna, albacore, bonito, young yellowtail, great amberjack, Spanish mackerel, and yellowtail^15^^)^. Based on the classification used in the Survey as mentioned above, these 19 species were classified into the 5 raw fish groups as follows: horse mackerel, sardine, mackerel, and saury: horse mackerel/sardine group; salmon and rainbow trout: salmon/trout group; flounder, red snapper, and cod: red snapper/flounder group; swordfish, bluefin tuna, big eye tuna, yellow fin tuna, albacore, and bonito: tuna/swordfish group; and young yellowtail, great amberjack, Spanish mackerel, and yellowtail: other raw fish group. Several continuous distributions were fitted to the concentration data of each fish group. In all fish groups, the statistics of Kolmogorov-Smirnov test and Anderson-Darling test were the lowest for a log-normal distribution and the resulting distributions were used for Monte Carlo simulations.

The methylmercury intake was probabilistically estimated using Crystal Ball (Oracle^©^, Redwood City, CA, USA) with a Monte Carlo simulation. Monte Carlo simulation is well known as one of the most powerful method to analyze complex problems which may occur on sceneries related with food safety and the estimated intakes using this method have been reported for a number of harmful substances^19^^-^^21^^)^. In the simulation we performed, in each trial, one methylmercury concentration and one amount of consumption in the corresponding group were randomly generated according to the distribution. The product of these 2 values was taken to be the intake from that group. The intake values from each of the 5 groups were totaled to arrive at the level of intake from raw fish and then divided by the body weight of 50 kg to determine average intake per kilogram body weight^22^^)^. A total of 100,000 Monte Carlo simulation iterations were performed.

## 3. Results and Discussion

### 3.1 Distribution of Methylmercury Concentrations

**Fig. 1.**
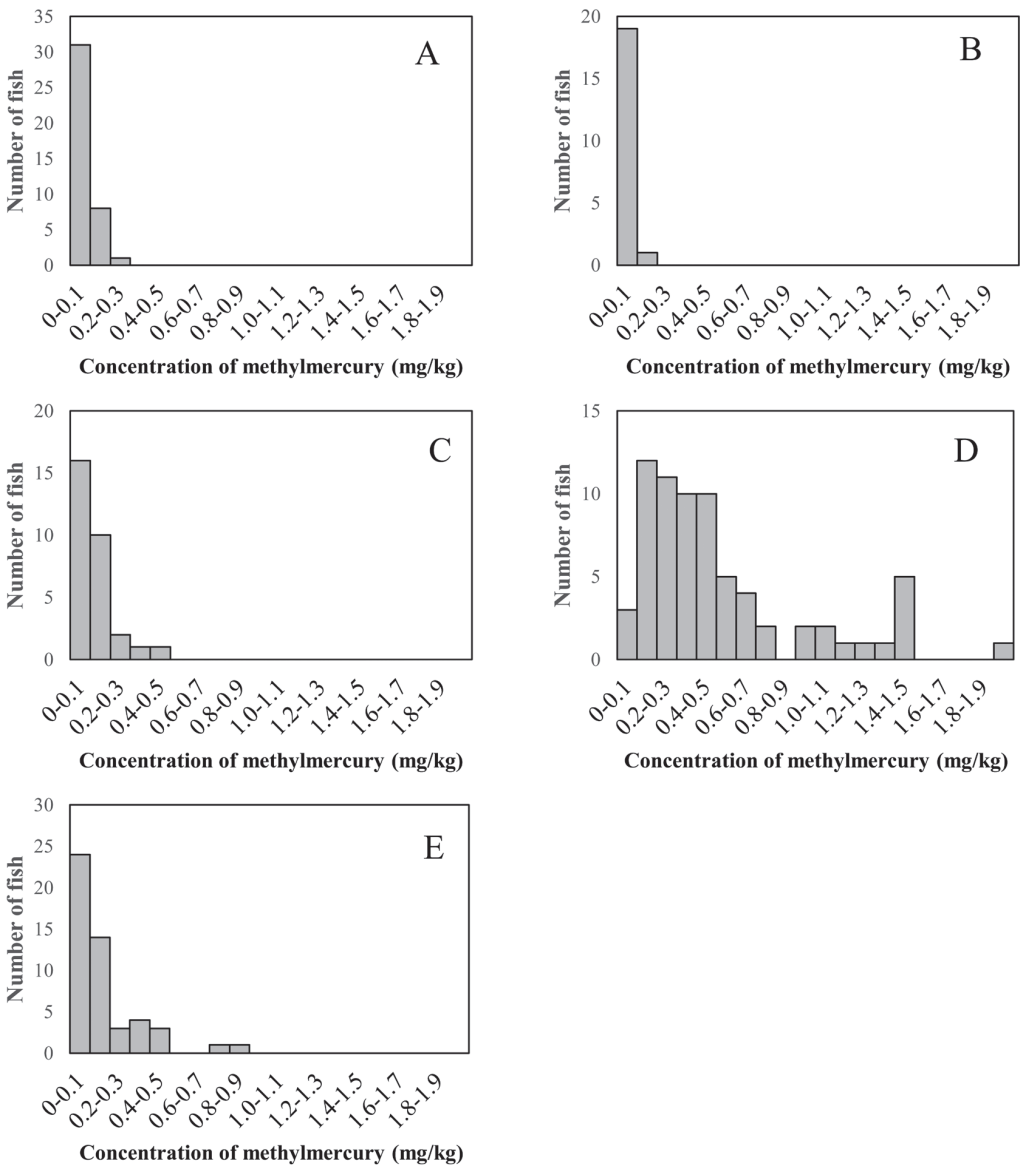
Observed distribution of methylmercury concentrations in 5 fish groups. A: Horse mackerel and sardine group, B: Salmon and trout group, C: Red snapper and flounder group, D: Tuna and swordfish group; E: Other raw fish group.

The distributions of methylmercury concentrations in the 5 raw fish groups are shown in **Fig. 1**. Methylmercury concentrations differed substantially across groups. The methylmercury concentrations of the samples of fish from the horse mackerel/sardine group were less than 0.3 mg/kg, and plotting the data in a histogram indicated that most concentrations were in the range 0.05-0.075 mg/kg. All samples from fish of the salmon/trout group had methylmercury concentrations less than 0.2 mg/kg, and the concentration was less than 0.05 mg/kg in 19 of the 20 samples. The methylmercury concentrations of samples of fish from the red snapper/flounder group were less than 0.5 mg/kg, and those in samples from the other raw fish group were less than 0.9 mg/kg. These samples contained slightly higher concentrations of methylmercury than the samples from the salmon/trout and horse mackerel/sardine groups. The methylmercury concentrations of some samples of fish from the tuna/swordfish group exceeded 1.0 mg/kg, with a maximum concentration of 1.9 mg/kg. Concentrations were higher than those in the other groups.

Histogram analyses of the groups revealed different concentration ranges, but all exhibited higher frequencies on the low side and tailed off on the high side. Since this shape indicated that methylmercury concentrations in fish do not follow a normal distribution, a log-normal distribution was fitted to the data. Means and standard deviations of the log-normal distributions of the groups are shown in Table 1. The mean concentration was highest in the tuna/swordfish group and lowest in the salmon/trout group. The mean concentration in the tuna/swordfish group was approximately 18-fold higher than that in the salmon/trout group. In each group, the standard deviation was comparable to or slightly larger than the mean.

**Table 1.** Mean and standard deviation of the log-normal distribution fitted to the distribution of the observed methylmercury concentration in 5 fish groups

Fish group	Horse mackerel and sardine	Salmon and trout	Red snapper and flounder	Tuna and swordfish	Other raw fish
Mean (mg/kg)	0.07	0.03	0.12	0.54	0.18
Standard deviation (mg/kg)	0.07	0.05	0.13	0.53	0.28

### 3.2 Distribution of Fish Consumption

**Fig. 2.**
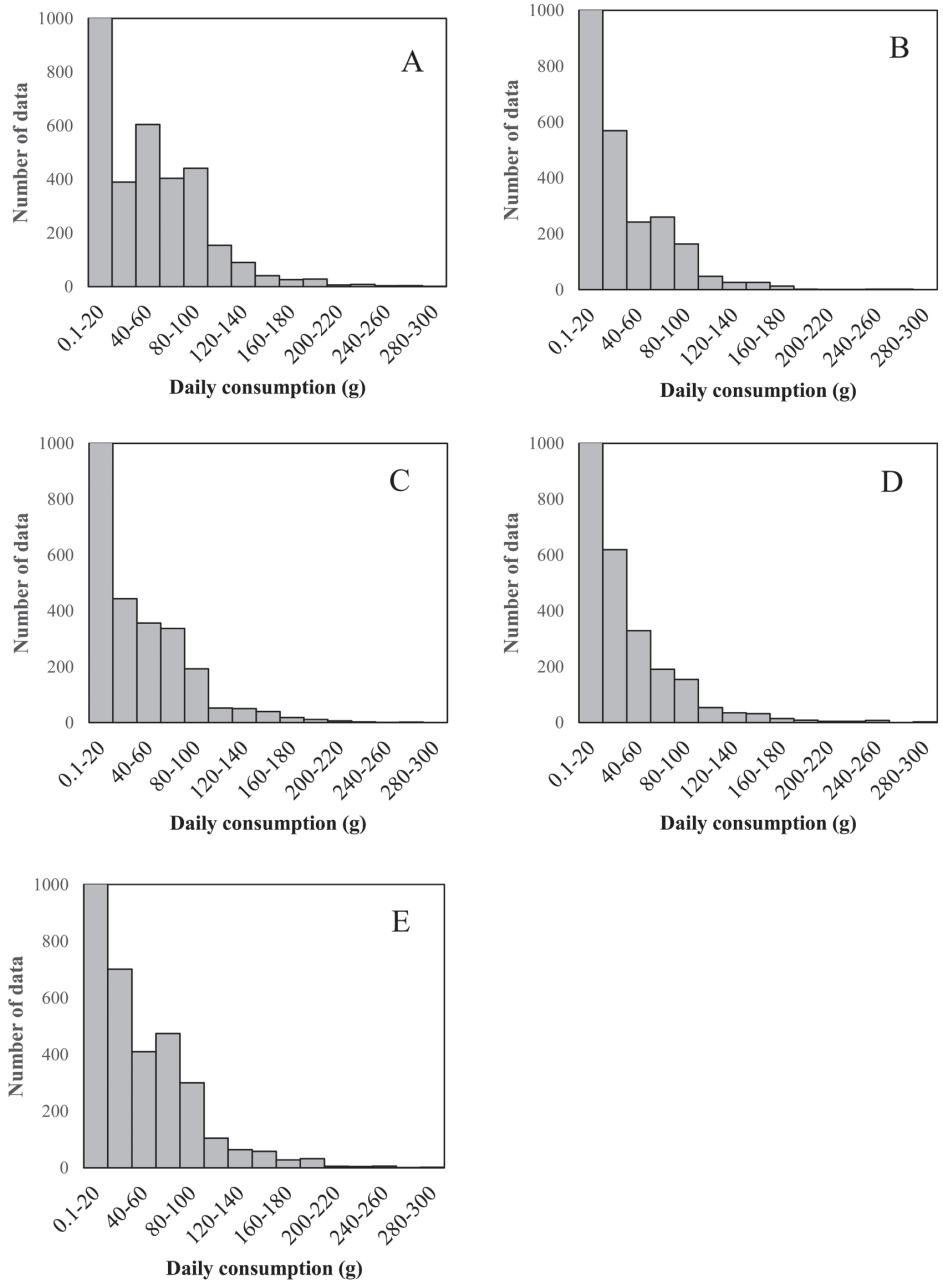
Distribution of daily consumption of fish from the 5 groups. A: Horse mackerel and sardine group, B: Salmon and trout group; C: Red snapper and flounder group, D: Tuna and swordfish group,E: Other raw fish group.

Distributions of the number of people consuming different amounts of fish in each of the different groups are shown in **Fig. 2**. The proportion of people consuming fish of the different groups on the day of the survey relative to the total number of people included in the survey was 17.5% in the horse mackerel/sardine group, 20.8% in the salmon/trout group, 25.2% in the red snapper/flounder group, 15.1% in the tuna/swordfish group, and 34.2% in the other raw fish group. Limiting the scope to the 1 day of the survey resulted in a very large proportion of people not consuming fish, so non-consumers were not included in the figures. Most of the people consumed no more than 20 g of the fish in each group, resulting in an overall left-skewed distribution. Others, albeit few, consumed 200 g or more. Fitting the data was impossible, as no continuous distribution conforms to this shape. Nevertheless, the data were well-suited to the simulation, as over 4,000 people consumed the fish of each group. The data were therefore used without fitting to any statistical distributions.

### 3.3 Evaluation of Scenario for the Simulation

Generating random numbers to represent methylmercury concentrations in the different fish according to a log-normal concentration distribution would result in unrealistically high concentrations with low probability. Using these random numbers in turn would result in unrealistic, very-high intake values. Such unrealistic concentrations also could make high values (such as the 95th percentile values) inaccurate. We therefore decided to perform simulations following three scenarios with different handling of an upper limit on generated methylmercury concentrations to evaluate the effects of this situation. In Scenario 1, no upper limit was established on methylmercury concentration. In Scenario 2, an upper limit on methylmercury concentration of 2 times the maximum concentration measured in each group was established. In Scenario 3, the maximum methylmercury concentration measured in each group was established as the upper limit.

**Fig. 3.**
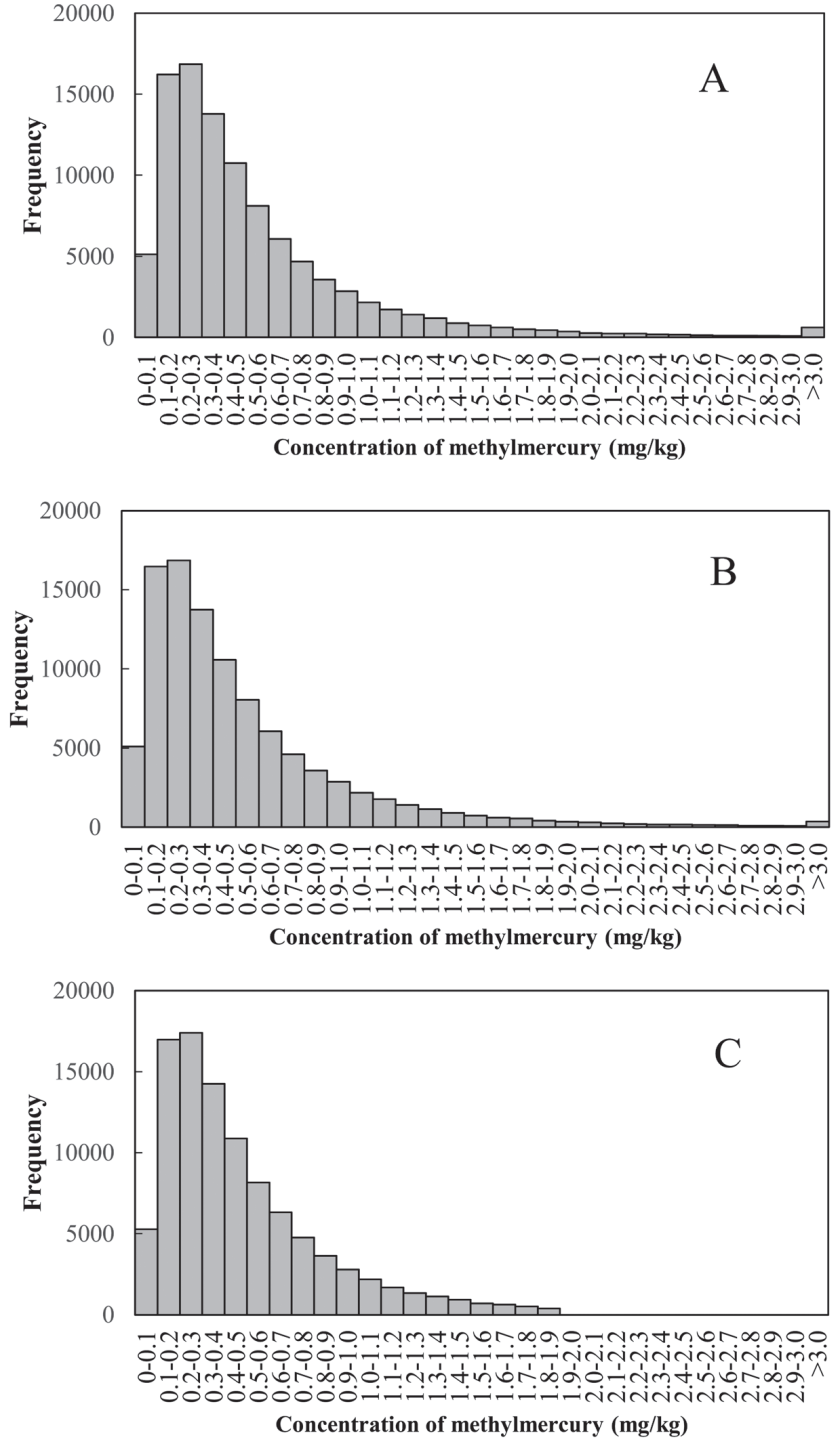
Distribution of random numbers of methylmercury concentration in the tuna and swordfish group. A: Scenario 1, B: Scenario 2, C: Scenario 3.

Distributions of the random numbers generated for the tuna/swordfish group under each scenario are shown in Fig. 3. These distributions, like the actual distributions of methylmercury concentrations shown in Fig. 1-D, exhibited the greatest frequency in the 0.1 mg/kg to 0.4 mg/kg range, but the distribution for Scenario 1 tailed extensively to the high end. In Scenario 1, which set no limits on the random numbers generated, a concentration of methylmercury approximately 5-fold higher than the actual maximum of 1.9 mg/kg was found in the tuna/swordfish group. Moreover, 0.025% of the random numbers were larger than this actual maximum. The shapes of the distribution following Scenario 1 and 2 were very similar, but the defined upper limit of the distribution of Scenario 2 was 3.8 mg/kg that was 2 times the maximum concentration measured.

Mean values, medians, 75th, 90th, 95th, and 99th percentiles of the distributions of methylmercury intake determined using Monte Carlo simulations for Scenarios 1 to 3 are shown in Table 2. Mean methylmercury intake was 0.097 µg/kg/day under Scenario 1, 0.093 µg/kg/day under Scenario 2, and 0.083 µg/kg/day under Scenario 3. The mean estimated intake under Scenario 3 was 0.86-fold of that under Scenario 1, which indicates that establishing an upper limit on the methylmercury concentration changes the mean. Evaluation of the ratios of the order statistics of Scenario 3 to Scenario 1 revealed that the median (50th percentile) was 0.96 and 75th percentile was 0.94, indicating that establishing an upper limit has less of an effect on these statistics than on the mean. The ratios, however, were 0.91 at the 90th percentile, 0.88 at the 95th percentile, and 0.83 at the 99th percentile. The effect of establishing an upper limit was more apparent at these percentiles. Scenario 3 used the actual maximum as the upper limit on methylmercury concentration. However, even the group with the most data points (tuna/swordfish group) had only 70 data points^16^^)^, indicating that using this maximum as the maximum concentration for the simulation would likely result in underestimation. Without a maximum, however, unrealistically high concentrations are theoretically possible. As mentioned before, a random number of approximately 5-fold higher than the actual maximum was generated after 100,000 iterations. Such a simulation inconsistent with actual concentration profiles could overestimate intake. The availability of much more concentration data would allow an appropriate upper concentration limit to be established, but currently available data are insufficient. We therefore established an upper limit on methylmercury concentration that was 2-times the actual maximum for the Scenario 2 simulation in this study. The statistics determined under Scenario 2 were, with the exception of the maximum, several percentage points lower than in Scenario 1. The results obtained under Scenario 2 simulation were used as the estimates for analysis in this study.

**Table 2.** Estimated statistical parameters of the daily methylmercury intake (µg/kg bw/day) distribution of Japanese general population

Simulation scenario	Scenario 1	Scenario 2	Scenario 3
Mean	0.0969	0.0929	0.0829
Median	0.0173	0.0169	0.0166
75 percentile	0.0792	0.0782	0.0746
90 percentile	0.231	0.228	0.211
95 percentile	0.419	0.410	0.366
99 percentile	1.19	1.15	0.99

### 3.4 Estimated Daily Intake

**Fig. 4.**
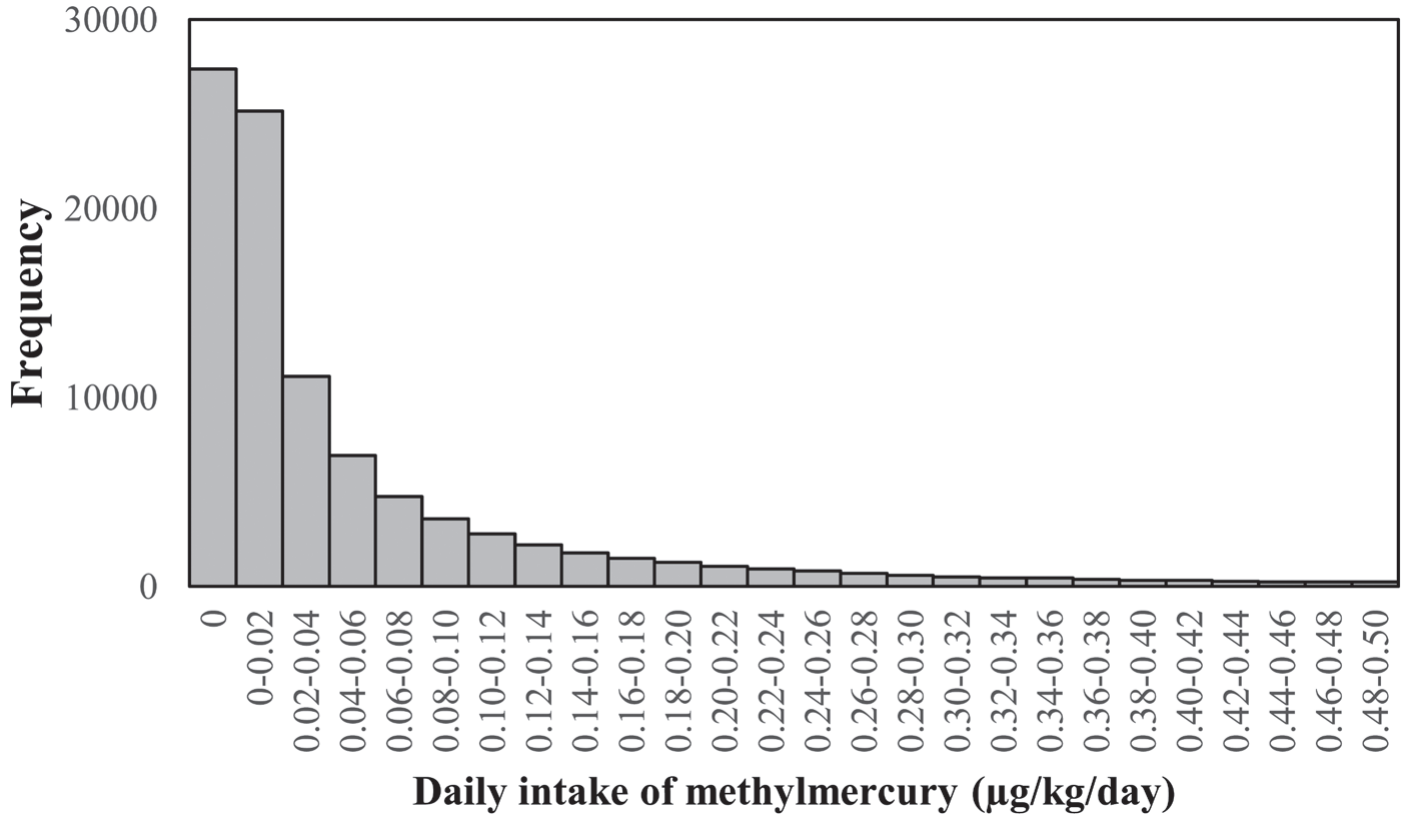
Distribution of the daily intake of methylmercury estimated via simulation.

The distribution of methylmercury intake determined under Scenario 2 is shown in **Fig. 4**. A methylmercury intake of 0 µg/kg bw/day was obtained in about 27% of the iterations. These estimates correspond to a situation in which no fish was consumed on the day in question. As both the methylmercury concentration and fish consumption exhibited downward-sloping distributions, the distribution of methylmercury intake also sloped downward. The proportions of methylmercury intake from 5 fish groups were 10% for horse mackerel/sardine group, 2% for salmon/trout group, 14% for red snapper/flounder group, 47% for tuna/swordfish group, and 27% for other raw fish group. Consumption of fish in the “other raw fish” group amounted to 41.4%, and consumption of fish in the tuna/swordfish group amounted to 22.7% of all fish consumed, indicating that consumption of fish in these groups contributed substantially to overall methylmercury intake from fish. Heavy consumption of fish in the “other raw fish” group relative to the other groups explains the large contribution of this group to methylmercury intake. The high concentrations of methylmercury in large predatory fish such as swordfish and tuna (tuna/swordfish group) and wild yellowtail (other raw fish group) explain the large contribution of the consumption of these fish species to overall methylmercury intake from fish.

As shown in Table 2, the mean daily intake of methylmercury estimated by the Monte Carlo simulation was 0.093 µg/kg bw/day. Zhang et al estimated the mean value of methylmercury intake for Japanese population to be 0.14 μg/kg bw/day by conducting a Monte Carlo simulation^23^^)^. The daily fish consumption was assumed to follow a log-normal distribution with the reported mean and SD and both raw fish and processed fish food products consumption were considered in their simulation. Our probabilistically determined estimate of mean methylmercury intake was about 70% of their estimate being close to the estimate of 0.132 µg/kg bw/day determined in a total-diet study^13^^)^. Our estimate was slightly lower than these two estimates (which included intake of processed fish food products) because it was calculated based on the simulation using methylmercury concentrations and consumption levels of raw fish only. The median intake was 0.017 µg/kg bw/day. The median was much lower than the mean because methylmercury intake was estimated to be 0 µg/kg bw/day in many of the iterations. The 90th percentile of our probabilistically determined methylmercury intake estimate was 0.228 µg/kg bw/day, and the 95th percentile was 0.410 µg/kg bw/day. The 95th percentile exceeded the TDI of 0.286 µg/kg bw/day calculated from the TWI established by the Food Safety Commission of Japan^6^^)^. The probability of a person taking in over 0.286 µg/kg bw of methylmercury on a given day was 7.6%. As shown in Table 3, each of the estimates for women, who are or may become pregnant, simulated under Scenario 2 was below the corresponding estimate for the Japanese general population. The 95th percentile was 0.305 µg/kg bw/day and exceeded the TDI as in the case of the general population.

**Table 3.** Estimated statistical parameters of the daily methylmercury intake (µg/kg bw/day) distribution of women (between the ages of 16 and 45)

Mean	Median	75 percentile	90 percentile	95 percentile	99 percentile
0.0699	0.0113	0.0568	0.169	0.305	0.87

The simulation-determined 95th percentile or the percentage of consumers exceeding the TWI is a single-day occurrence. TWI is set considering life-long intake; therefore methylmercury should not be lightly classified as a health risk based on one day of intake. However, if a population that consumes large amounts of fish (right side of Fig. 2) also consumes fish frequently, this population would have a higher likelihood of taking in methylmercury in excess of the TWI than would a population that consumes an average amount of fish at an average frequency. A simulation of intake in the former population would require data indicative of dietary habits, such as data resulting from a food consumption survey conducted over multiple days.
